# Leishmania (Viannia) guyanensis Causing Localized Cutaneous Leishmaniasis in a Traveler

**DOI:** 10.7759/cureus.27055

**Published:** 2022-07-20

**Authors:** Ian K Motie, John Sia, Katherine Burns, Natan Kraitman, Roberto Mercado

**Affiliations:** 1 Internal Medicine, Sarasota Memorial Hospital, Sarasota, USA; 2 Internal Medicine, Florida State University College of Medicine, Sarasota Memorial Hospital, Sarasota, USA; 3 Infectious Disease, Sarasota Memorial Hospital, Sarasota, USA

**Keywords:** tropical disease, vector borne disease, parasite infection, leishmania guyanensis, cutaneous leishmaniasis

## Abstract

We report on a 66-year-old male who presented for evaluation of rapidly expanding lesions on his lower extremities. He first noticed these lesions following a trip to Costa Rica, in which he was bitten by several unidentified bugs. He was initially treated empirically with antibiotics in the outpatient setting with no improvement of his symptoms. His lesions continued to expand and spread locally which prompted further workup with a biopsy of one of his lesions. He was ultimately found to have *Leishmaniasis (Viannis) guyanensis *confirmed by microscopy and polymerase chain reaction. He was treated with aggressive wound care and amphotericin B due to the risk of progressing to involve his mucosa.

## Introduction

Leishmaniasis encompasses a broad spectrum of diseases caused by a large group of protozoa within the genus *Leishmania *[1]*. *It is transmitted by the bite of phlebotomine sand flies [1]. The geographic distribution and presentation of disease often depend on the specific species: Old World *Leishmania *and New World *Leishmania* [2]. Old World leishmaniasis is primarily identified in Asia, the Middle East, tropical regions of Africa, and southern Europe [1,2]. New World leishmaniasis is primarily identified in Mexico, Central America, and South America [1,2]. 

These are also further differentiated between cutaneous disease and visceral disease. Disease manifestation depends on the causative leishmanial species, although there can be some overlap between species [2]. Visceral leishmaniasis can be a more severe manifestation but often does not present with cutaneous findings [2]. Visceral leishmaniasis primarily affects the liver, spleen, and bone marrow with clinical manifestations of fever, weight loss, hepatosplenomegaly, and pancytopenia [1-3].

Cutaneous leishmaniasis itself consists of a spectrum of disease presentations which consist of localized cutaneous leishmaniasis, diffuse cutaneous leishmaniasis, and mucosal leishmaniasis [1-3]. In this case, we discuss the presentation of *Leishmania (Viannis) guyanensis*, one of the species known to present with mucocutaneous disease, and our approach to both diagnosis and treatment.

## Case presentation

A 66-year-old male presented to the hospital after he developed cutaneous lesions several months prior to arrival, despite outpatient antibiotics. The patient states that he first noticed small lesions on his lower extremities approximately four months prior to presentation. These lesions were first noticed in November 2021 following several insect bites. The following day, the patient noticed a “blackhead” at the site of the insect bites, which he was able to manually extract. In the following days, he noticed several other lesions involving his lower extremities. The lesions were initially small and associated with mild erythema but increased in size over the next three months and darkened in color. Additionally, he began developing smaller satellite lesions nearby on both of his knees and thighs.

Because his wounds continued to progress, he sought care in the outpatient setting approximately six weeks prior to our evaluation. At that time, he was treated empirically with a one-week course of amoxicillin/clavulanate 875 mg/125 mg, which did not provide any resolution of symptoms. He again sought care at a separate institution and underwent a biopsy of one of his lesions, where he was then placed on doxycycline 200 mg daily for approximately three weeks. This initially provided some benefit, but his lesions continued to expand and gradually became painful, which ultimately led to his current presentation.

On further history, the patient reports traveling in 2021 to Costa Rica, Brazil, Italy, Greece, Peru, and Costa Rica again in November 2021. Of note, the patient developed these lesions in November 2021 after incurring a bug bite while in Costa Rica. He was then exposed to river water in Costa Rica immediately after the bug bite. He reports that during his trips to Costa Rica, he noticed several similar lesions affecting local Costa Ricans during his time there.

**Figure 1:**
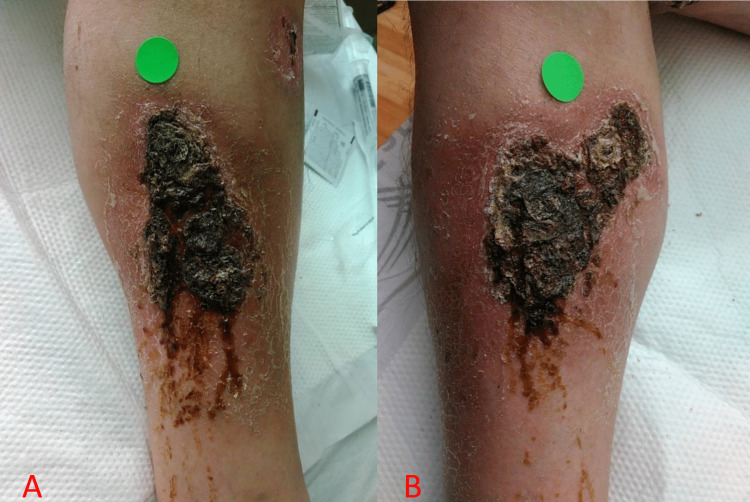
Lesion located on the medial aspect of the left calf (A) and right calf (B)

On physical examination, there were multiple lesions on his bilateral lower extremities that appeared to have a thick, hyperkeratotic crust with surrounding erythema and tenderness to palpation. There was a significant centralized eschar noted in larger lesions. The two largest lesions were present on his left calf, measuring approximately 7 cm x 5 cm, and his right calf, measuring approximately 7 cm x 6 cm (Figure 1). There were no other lesions present elsewhere on the patient’s body. The remainder of his examination was otherwise normal.

**Figure 2:**
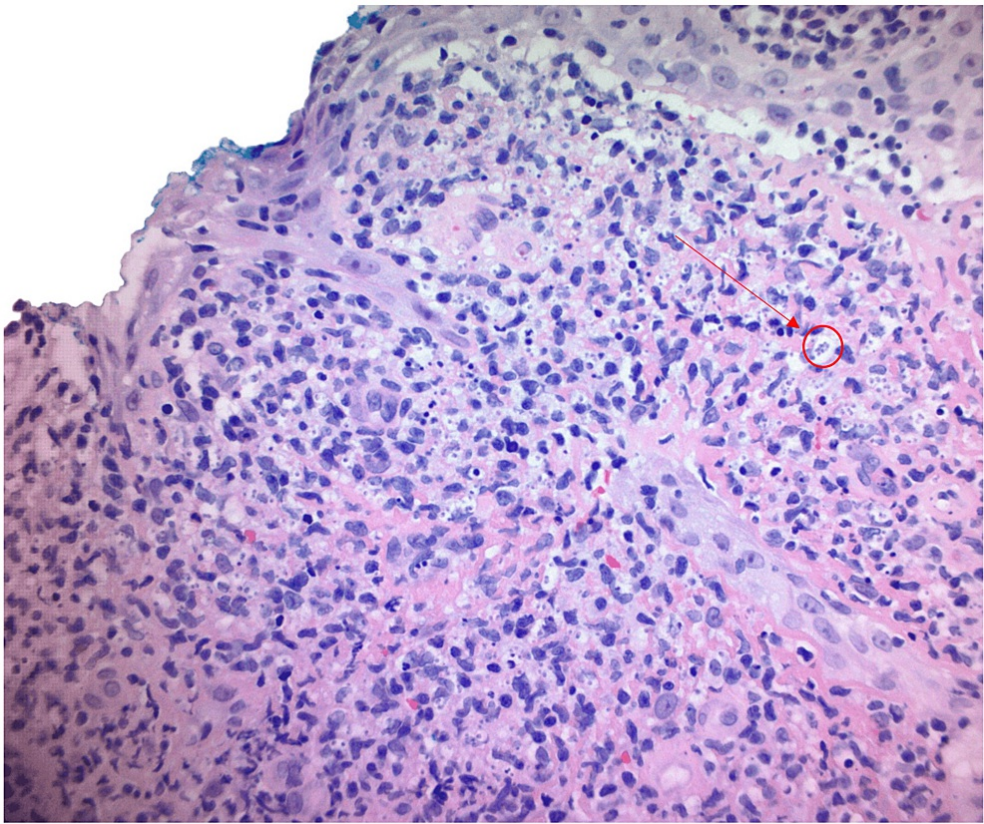
Histopathology of the biopsied lesion at 400x magnification demonstrating amastigotes circled in red

The results of his biopsy obtained at a separate facility showed visible amastigotes and were consistent with leishmaniasis (Figure 2). Surface wound cultures obtained on presentation showed scant growth of *Streptococcus agalactiae*. Due to the risk of progressive mucosal involvement, the patient underwent polymerase chain reaction (PCR) testing, and his cultures were further analyzed at the University of Washington. They were confirmed to be secondary to *Leishmania (Viannis) guyanensis*. Upon identification of the causative organisms, the patient was treated with seven days of liposomal amphotericin B for cutaneous leishmaniasis and ceftriaxone for a presumed superimposed *Streptococcus agalactiae* bacterial infection. After completing inpatient antimicrobial therapy, he was discharged home with home wound care and instructed to follow up with the hospital’s wound care clinic and infectious disease service. He has since followed up with outpatient wound care and with lesions showing interval improvement.

## Discussion

Leishmaniasis is not commonly encountered in the United States as it is more endemic in the tropics and subtropics; however, our patient’s presentation with localized lesions and significant travel history helped to narrow down his differential diagnosis towards an etiology related to exposure.

In consideration of cutaneous leishmaniasis, the patient had the appropriate travel history and environmental exposure. The mouth parts of the disease vector sand fly consist of six knife-like stylets but are not equipped to penetrate clothing which causes the disease to only affect exposed areas of the body [1]. In our patient, all lesions were confined to his lower extremities, which were likely exposed during his travel. The incubation period for cutaneous leishmaniasis can range from weeks to months, with several of the countries visited by our patient known to be endemic for different species causing leishmaniasis [3]. Transmission of cutaneous leishmaniasis occurring human-to-human is not fully understood or studied but does not appear to occur nearly as often as vector-borne transmission [4].

If concerned for leishmaniasis, tissue samples should be obtained and demonstrate parasitic activity in the specimen [1]. This should be done by removing the hyperkeratotic tissue and obtaining a full-thickness punch biopsy to fully assess histology and any cellular response. PCR is also shown to be the most sensitive form of testing for leishmaniasis and can help provide speciation of the organism [5]. Leishmaniasis is reported to the Centers for Disease Control and Prevention (CDC) once confirmed. For our patient, his biopsy showed amastigotes, the form of parasite found within cells that multiply, ultimately infecting other mononuclear phagocytic cells. Serologic testing for Leishmaniasis antibodies are less reliable when attempting to diagnose leishmaniasis and are less useful when compared to the previously mentioned interventions [6].

Approaching treatment of cutaneous leishmaniasis first assesses the risk of mucosal disease. Our patient’s species of *Leishmania (Viannis) guyanensis *is known to cause cutaneous manifestations but has also been identified as a causative agent of mucosal disease [7]. Mucosal leishmaniasis often presents with an erosion of the nose, mouth, or nasal septum, often leading to subsequent disfigurement. This form can include a more severe presentation that involves the lower respiratory system resulting in respiratory distress [8]. As such, immediate treatment is imperative to prevent the risk of progression. Systemic agents approved for use include miltefosine, azoles, amphotericin deoxycholate, and liposomal amphotericin [9]. Any eschars should be debrided and treated with local wound care to maximize recovery. Superimposed bacterial infections are also common and should be treated if present.

## Conclusions

Cutaneous leishmaniasis should be suspected in patients from countries where the disease is endemic or those who are otherwise healthy with recent travel to endemic areas, including tropical and subtropic climates. Initial workup relies on a thorough physical examination to evaluate for localized extent of disease or mucosal involvement. Leishmaniasis is confirmed with a punch biopsy of an active lesion. Further studies, such as PCR, can assist with the speciation of the parasite. Once confirmed, this should be reported to the CDC and treated with localized wound care or systemic therapy if there is a concern for mucosal involvement based on the species. Close follow-up with wound care and infectious disease specialists is also imperative to assess response to therapy. Patients can avoid contracting this disease by covering all exposed areas of the body with clothing to prevent bites from the phlebotomine sand flies which transmit the disease. Patients should also be counseled that current evidence does not suggest that they can transmit the disease via human-to-human transmission. 
